# What makes eyespots intimidating–the importance of pairedness

**DOI:** 10.1186/s12862-015-0307-3

**Published:** 2015-03-09

**Authors:** Ritwika Mukherjee, Ullasa Kodandaramaiah

**Affiliations:** School of Biology, Indian Institute of Science Education and Research Thiruvananthapuram, CET campus, Trivandrum, 695016 India

**Keywords:** Eyespots, Conspicuousness, Eye-mimicry, Intimidation, Startle display, Junonia almana

## Abstract

**Background:**

Many butterflies possess striking structures called eyespots on their wings, and several studies have sought to understand the selective forces that have shaped their evolution. Work over the last decade has shown that a major function of eyespots is their ability to reduce predation by being intimidating to attacking predators. Two competing hypotheses seek to explain the cause of intimidation, one suggesting ‘eye-mimicry’ and the other their ‘conspicuousness’ as the reason. There is an on-going debate about which of these better explains the effectiveness of eyespots against predation. We undertook a series of indoor experiments to understand the relative importance of conspicuousness and eye-mimicry, and therefore how predator perception may have influenced the evolution of eyespots. We conducted choice tests where artificial paper models mimicking *Junonia almana* butterflies were presented to chickens and their preference of attack recorded.

**Results:**

We first established that birds avoided models with a pair of eyespots. However, contrary to previous, outdoor experiments, we found that the total area of eyespots did not affect their effectiveness. Non-eye-like, fan shaped patterns derived from eyespots were found to be just as effective as eye-like circular patterns. Furthermore, we did not find a significant effect of symmetry of patterns, again in discordance with previous work. However, across all experiments, models with a pair of patterns, symmetric or asymmetric, eyelike or non-eye-like, suffered from fewer attacks compared with other models.

**Conclusions:**

The study highlights the importance of pairedness of eyespots, and supports the hypothesis that two is a biologically significant number that is important in prey–predator signalling. We discuss the implications of our results for the understanding of eyespot evolution.

**Electronic supplementary material:**

The online version of this article (doi:10.1186/s12862-015-0307-3) contains supplementary material, which is available to authorized users.

## Background

Eyespots - conspicuous, circular or quasi-circular colour markings – are remarkably common morphological features in the animal kingdom. They are widely found in insect groups, especially butterflies and moths, as well as in vertebrates such as fish, birds and frogs [1-3]. They exhibit enormous diversity in morphology, occurring in an array of sizes and colour combinations, from very simple to highly complex structures. Biologists have long been fascinated by these eyespots and multiples lines of investigation have addressed the question of how and why eyespots have evolved in different groups of organisms. These include evo-devo [4-16], behavioral [1,2,17-27], phylogenetic [28-31] and theoretical [32] studies. Although occasional studies were carried out since the early 1900s, the last few years have culminated in a range of novel studies, which have significantly furthered our understanding of the evolution of these intriguing structures, and eyespots have become a very exciting model system in evolutionary and developmental biology [32-34].

Lepidopteran insects have been especially popular for investigations into eyespot evolution. Major strides have been taken toward understanding the genetics and developmental processes involved in eyespot formation in butterflies, particularly through studies on laboratory populations of *Bicyclus anynana* [6,35-40]. Comparative studies within a phylogenetic framework have shed light on the patterns of evolution of eyespots and furthered our understanding of the evolutionary forces that may have shaped the multitude of eyespots [9,28-31]. Experimental studies have directly demonstrated the wide range of selective forces that are likely to have shaped the myriad eyespots currently found in nature. Investigations have shown that eyespots in *B. anynana* are used as signals in assessing the quality of mates by both males and females [41-43]. Eyespots are also implicated as signals in the context of male-male competition in fish [44]. However, the most widespread selective agent shaping the evolution of eyespot is, arguably, predation pressure.

Two broad hypotheses explain how eyespots may be effective against predation. Large, conspicuous eyespots are considered to be intimidating for the predator thereby decreasing the chances of attack – the “Intimidation Hypothesis”, whereas smaller eyespots closer to the wing margin are thought to attract attention toward themselves thus deflecting predatory attacks away from the more vital parts of the prey – the “Deflection Hypothesis” [3,45-48]. There is experimental evidence supporting both hypotheses (intimidation hypothesis [2,22,23,25,26]; deflection hypothesis [20,49-51]). The current study focusses on the intimidation effects of large, conspicuous eyespots, and addresses how predator perception influences eyespot evolution.

### Intimidation hypothesis

Blest’s pioneer work [1] demonstrating the role of eyespots in reducing predation paved the way for further intensive studies establishing that eyespots can indeed intimidate predators. Eyespots are not only effective in intimidating the predator when displayed suddenly, as in the case of peacock butterflies (*Inachis io)* and eyed hawk-moths (*Smerinthus ocellata),* thereby startling the predator [1,2,52], but also when constantly displayed, inducing predators to abort or delay their attack [25,34,53]. For instance, studies based on artificial models with various eyespot-like designs printed on them [22], as well as those based on real butterflies [25,26], have shown that the constant display of eyespots can reduce attacks. Therefore, movement of wings or other eyespot bearing structures is not a pre-requisite for intimidation.

### Why do eyespots intimidate predators?

The most commonly suggested and cited explanation for the intimidating effect of eyespots is the ‘Eye-mimicry hypothesis’ [1] wherein eyespots are thought to instil fear or aversion in predators by resembling the eyes of their own enemies. Although the intimidating effect of eyespots has been shown in multiple studies, there is still a lack of a deeper understanding of the mechanistic basis of “why” they intimidate [2,48,52,54]. Surmising that eyespots’ resemblance to vertebrate eyes is an anthropomorphised and subjective assumption, Stevens and colleagues argued that eyespots may be effective simply because of their conspicuous and contrasting features – the ‘Conspicuousness hypothesis’ [3,34]. They performed a series of well-designed experiments, which have strongly supported the importance of conspicuousness. Using artificial paper models with a mealworm in the centre, they first demonstrated that highly contrasting features such as bars, triangles and squares were also effective in deterring predation in addition to circular, eyespot-like structures [53]. The total area of the conspicuous signal was found to be most important, rather than the number of eyespots or the size of individual eyespots. Thus, one or three eyespots were equally effective compared to a pair of eyespots, as long as the total area of the eyespots was conserved, suggesting that resemblance to a pair of vertebrate eyes is inconsequential. They further found that markings with centres displaced inwards, making them seemingly three-dimensional and eye-like, were attacked no differently than non-eye-like markings without centre displacement [22]. They went on to conduct another study where they compared the effectiveness of eyespots with a black pupil surrounded by a yellow ring, which most closely resembles vertebrate eyes, with eyespots having other colour combinations. Their study indicated that several other non-eye-like colour combinations were as effective as black and yellow [22,34,53].

Eyespots on the dorsal wing surface of lepidopterans are bilaterally placed in nature. Based on laboratory experiments using chickens and artificial prey, Forsman and colleagues [55] found that symmetry in size, shape and colour patterns is crucial for averting predatory attacks, thus indirectly supporting the eye-mimicry hypothesis. However, consequent studies investigating the same in a natural environment provided no significant support for the importance of symmetry [21]. Lepidopteran eyespots commonly bear a small structure called a ‘sparkle’ on the upper part of the eyespots’ pupil. The ‘sparkle’ is suggested to mimic the natural corneal total light reflection, thus thought to create an illusion of an ‘eye’ for UV-sensitive birds. Blut and colleagues [23] conducted field experiments, demonstrating that eyespots with the ‘sparkle’ in a natural position induce stronger aversion than eyespots with the ‘sparkle’ in an unnatural position. Thus the presence of the ‘sparkle’ is suggested to reinforce the eye-mimicry hypothesis.

In summary, the question of which of the two hypotheses better explains the evolutionary significance of the eyespots in an anti-predatory role is yet to be conclusively answered. Studies so far that have attempted to answer this question have largely relied on outdoor experiments with simplistic, artificial models that do not necessarily resemble naturally occurring eyespots. The aim of the current study was to understand how the spatial distribution, number, size and symmetry of eyespots affect the effectiveness of their visual threat. To discriminate between the two competing hypotheses, a series of experiments were conducted testing the effectiveness of the eyespots against predation by domestic chickens *Gallus gallus domesticus* by presenting them with paper targets mimicking the peacock pansy butterfly *Junonia almana.*

## Results

Although the birds had varied behavioural responses on being presented with the artificial models, most of them eventually attacked both the models. Despite the large sample size in every experiment, all birds pecked only at the broken corn and never on the signalling patterns or the wings.

### Experiment 1

**Figure 1:**
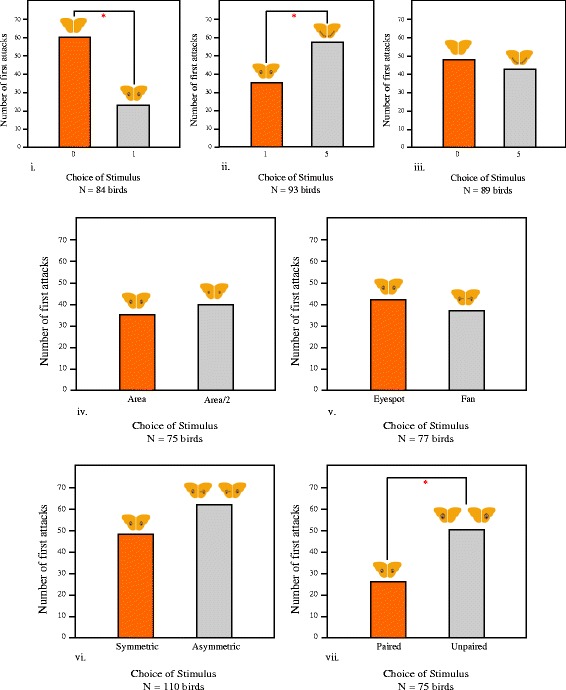
**Barplots illustrating the number of first attacks on models used in the 5 experiments.** An asterisk indicates significance (P <0.05) **i.** The first test in experiment 1: no eyespots *vs* one eyespot/hindwing. **ii.** The second test in experiment 1: 1 eyespot *vs* 5 eyespots/hindwing **iii.** The third test in experiment 1: no eyespot *vs* 5 eyespots/hindwing **iv.** Second experiment; natural sized eyespots *vs* eyespots with half the area **v.** Third experiment: natural eyespots *vs* fan-like eyespots **vi.** Fourth experiment: symmetric eyespots *vs* asymmetric eyesopts **vii.** Fifth experiment: paired *vs* unpaired single eyespot.

The first experiment consisted of three trials employing a total of 266 birds. Each bird was presented a pair of prey items, and no bird was used more than once. In the first test (0 *vs* 1 eyespot per wing), birds significantly preferred to first attack the eyespot-less model over the eyespotted model (Binomial Test: N = 84; P = 0.0001) (Figure 1 i.), with a significantly higher attack latency for the eyespotted model (Wilcoxon signed-rank test: V = 798; P = 0.000011; N = 84). In the second test (1 *vs* 5 eyespots per wing, where the area of each eyespot in the latter treatment was reduced such that the total eyespot size was equal in both treatments), birds preferred to first attack the model with 5 eyespots (Binomial Test: N = 93; P = 0.0220) (Figure 1 ii.), taking significantly lesser time to attack the one with 5 eyespots (Wilcoxon signed-rank test: V = 2716; P = 0.0422; N = 93). In the third test (0 *vs* 5 eyespots per wing) both models suffered similar rates of first attacks (N = 89; P = 0.5250) (Figure 1 iii.) and attack latency (V = 1820; P = 0.4559; N = 89).

**Figure 2:**
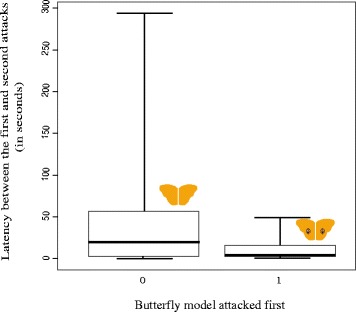
**Boxplot describing the latency between the first and second attack (in seconds) of the birds.** The dark horizontal line within the box represents the median with the box denoting the first and third quartiles and the whiskers being the minimum and maximum values observed. Upon being presented with no *vs* 1 eyespot per hindwing (No eyespots: m = 20 s, IQR = 51 s; 1 eyespot/hindwing: m = 5.5 s, IQR = 11.25 s), the latency was significantly higher when the birds attacked the model with 1 eyespot/hindwing after attacking model with none (Mann–Whitney *U* Test: W = 942, P = 0.0276).

In the first test, birds that preferred to attack the eyespot-less model first, attacked the second model (with 1 eyespot per wing) after a significantly longer time than birds that attacked the models in a reverse order (Mann–Whitney *U* Test: W = 942, P = 0.0276) (Figure 2). However, this difference between treatments in terms of duration from first to second attacks was not significant in the next two tests (1 *vs* 5 eyespots/hindwing: W = 1005, P = 0.9367; 0 *vs* 5 eyespots/hindwing: W = 1009, P = 0.8361).

### Experiment 2

Birds showed no preference between models with a pair of natural sized eyespots and those with a pair of smaller eyespots (N = 75; P = 0.6445) (Figure 1 iv.). The time taken to attack either of the models did not differ (V = 1406; P = 0.9220; N = 75). Furthermore, there was no significant difference in the latency between the first attack and the second, depending on which model was attacked first (W = 586, P = 0.2225).

### Experiment 3

Birds responded similarly to models with a pair of natural eyespots and those with fans (N = 77; P = 0.6488) (Figure 1 v.). Moreover, there was no difference in the attack latency for either treatment (V = 1416.5; P = 0.6674; N = 77) and in the duration between first and second attacks with either model being attacked first (W = 750.5, P = 0.8978).

### Experiment 4

Results indicated no difference in the reaction of the birds to models with a pair of natural eyespots and those with one eyespot and one fan (N = 110; P = 0.2150) (Figure 1 vi.). Neither was any difference observed in the time taken for attacking either choice (V = 3585.5; P = 0.1119; N = 110). Comparison of the attack latency between the first attack and the second with either of the model being the first choice gave no significant results (W = 1632.5, P = 0.3817).

### Experiment 5

Birds attacked models with a single eyespot significantly more than those with a pair of eyespots (N = 75; P = 0.0052) (Figure 1 vii.), taking a longer time to attack the models with a pair of patterns (V = 1877; P = 0.0170; N = 75). However, the latency between the first attack and the second when either of the models was the first choice did not differ significantly (W = 602, P = 0.7955).

## Discussion

**Figure 3:**
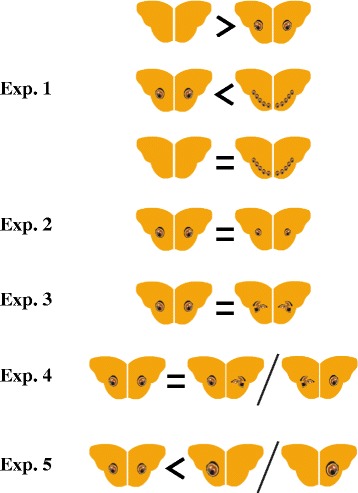
**Summary of the preferences for first attacks of birds in all the experiments described previously.** ' > ' and ' < ' symbols indicates the significant preference and ' = ' indicates no difference.

In the series of experiments employing a total of 603 birds, we have tested specific predictions of the eye-mimicry and conspicuousness hypotheses, two competing hypotheses that have a bearing on our understanding of how eyespots may have evolved in nature. The data do not unequivocally favor either hypothesis. Figure 3 summarizes results from all experiments. However, the results of our study question the generality of existing paradigms and augment our understanding of what properties make eyespots effective against predation.

In the first experiment, models with a pair of eyespots were attacked fewer times than the eyespot-less model. Birds also took a relatively longer time to attack the model with a pair of eyespots. These results strongly corroborate the intimidation hypothesis. However 10 eyespots with the same total area as the pair of eyespots did not confer significantly different protection compared to the spotless models.

The second experiment explicitly tested the effect of the size of the signal, where birds were offered a choice between a pair of natural sized eyespots and a pair of smaller eyespots. The results showed no significant difference in predatory attacks between models, and birds attacked both models equally quickly. Stevens et al. [22] found that the total area of the conspicuous signal determines eyespots’ effectiveness, irrespective of the number of eyespots (1, 2 or 3). They hence concluded that their data support the conspicuousness hypothesis, which predicts intimidation to increase with salience of the signal. Results of our first two experiments are in marked contrast, and provide no support for this prediction. In Experiment 5, where a single large eyespot was pitted against a pair of eyespots with the same total area, models with smaller eyespots experienced lesser predation, again in discord with findings of Stevens et al. [22].

A previous study [26] using wings of real butterflies and birds as predators, reported no difference in latency to attack between prey with four and two approximately equally sized eyespots. We note that both studies that have not found an effect of size or total area have employed real eyespots with a single bird species in controlled, indoor experiments, whereas an effect of size was found only in the outdoor experiments with highly simplified patterns and where the effect observed is that of the predator community composed of unknown predators. Further studies are needed to understand under what conditions and predatory systems the total area of eyespots makes a significant difference.

The third experiment explicitly investigated the importance of structural resemblance to eyes for deterrence. We found that models with a pair of eyespots and a pair of fans suffered similar number of attacks. With both signals being equally conspicuous, the eye-mimicry hypothesis predicts that the effectiveness of eyespots will be reduced once the shape of the eye is lost. Therefore, data from this experiment do not support the eye-mimicry hypothesis.

The results of the fourth experiment indicate that shape asymmetry does not hinder the effectiveness of eyespots. Results from previous studies that tested the effect of asymmetry have been mixed. A negative effect of asymmetry was found in Forsman et al.’s [55] study, where artificial prey were presented to chicken. However, field experiments by Stevens et al. [21] demonstrated no effect of asymmetry. Given these confounding results, more work is needed to understand the relevance of symmetry of eyespot-like patterns.

The fan-like structures used in the current study were made from cut-out sectors of the original circular eyespot, which might have decreased their possibility to appear markedly different from the original ones. Furthermore, many birds took a very short time (typically less than a second) to peck on the food once they had sighted it. This could possibly result in “blurring” of the patterns. Thus we cannot discount the possibility of the differences between the eyespots and fans being too subtle to evoke different reactions from birds.

Interestingly, in the first four experiments, all models that rendered effective deterrence possessed a pair of stimuli, eyelike or non-eyelike, arranged bilaterally symmetrically on the two wings. The last experiment was aimed to determine if being in a pair is pivotal. Indeed, the results showed that birds avoided the model with paired signals more often than they did a single, large eyespot. Scaife [56] reported that birds appeared to avoid paired, eye-like stimuli more than they did a single stimulus. However, all stimuli in his experiment were equally sized, and therefore the former had a larger total stimulating area. In a subsequent experiment, Jones [57] found that two eyelike stimuli elicited more avoidance compared to one or three stimuli of the same size, and concluded that pairedness of eye-like stimuli is critical, perhaps by ‘completing the eye-gestalt’. Jones and other authors [57-59] have argued that two is a biologically significant number in pattern signalling. Although recent studies by Stevens and colleagues [22] have contradicted this, our study strongly supports the importance of pairedness of conspicuous eyespot-like patterns for deterrence.

We stress that we have only tested asymmetry in shape, but not positional asymmetry. For example, the birds may respond differently to two eyespots which are not centered together on the same latitudinal or longitudinal axis. Furthermore, the influence of the position of an eyespot pair relative to the body will be worth investigating.

### Latency to attack the second model after the first

In Experiment 1, where spotless models were compared against models with a pair of natural eyespots, the duration between first and second attacks was greater when the second model had eyespots, indicating that eyespots increased the hesitation to attack. The remaining tests (all comparing models with conspicuous patterns) demonstrated no significant differences in latency to attack the second model (Additional file 1: Figure S2). We surmise that once a bird encounters eyespots, it will become more hesitant to attack, at least momentarily. This is corroborated by our visual observations on the behaviour of birds during trials.

### Implications for the evolution of eyespots

The current study is the first study to demonstrate that artificial, eyespot-like structures can reduce predation as a result of innate aversion by birds. Chicks used in the study had been reared from birth in the poultry farm and hence naive in terms of exposure to predators. It follows that the aversion towards eyespot-like structures is innate, rather than based on learning and association of eyespots with danger. Another study that used naive laboratory reared birds has reported aversion to eyespots, but those found on real butterfly wings [26].

Although our data are seemingly in support of the eye-mimicry hypothesis, we stress that we have found no strong evidence for either hypothesis. We surmise that conspicuousness, while being very important under many conditions, is not necessarily the single most important factor determining the effectiveness of eyespots. The results suggest that resemblance to a pair of eyes enhances the effect of conspicuous stimuli much more than the total area (or size) does. However, the structural resemblance of individual patterns to a vertebrate eye need not be perfect.

Our study has implications for the evolution of eyespots from the perspectives of development and predator perception. Large, presumably intimidating, eyespots are more abundant on the dorsal wing surfaces in many lepidopteran groups such as *Junonia* and *Bicyclus* [1,28]. Developmentally, a pair of intimidating eyespots may be easier to evolve on the dorsal surface of butterflies since both pairs of wings (i.e. left and right) are visible to the predator when the wings are held open. Therefore, the evolution of a single eyespot on one wing on the dorsal surface (either hindwing or forewing) results in a symmetric pair of eyespots (on either side of the body), which could significantly enhance the effectiveness of eyespots. When the butterfly rests with wings held together, the ventral surface of a single pair of wings (a forewing and a hindwing) are visible, making it relatively more difficult for a pair of large eyespots to evolve on the ventral wing surface.

Furthermore, it is plausible that complex eyespots started evolving as much simpler markings on the dorsal surface. Even imperfect initial markings could have provided some benefits by being present as a pair, as is suggested by Experiment 3, thereby compensating for any cost in terms of increased risk of being detected by predators. The critical question of why highly intricate, apparently three-dimensional, eyespots such as those found in *Junonia* have evolved remains incompletely answered. Perhaps these eyespots are also used as signals in communication between sexes, or in the context of intra-sexual conflict.

Although the results in our study agree with previous work [26], they clearly conflict with aspects of some other studies [22,34,53], highlighting the importance of receiver bias (i.e. effect of type of predator) and the environmental context. Experiments by Stevens and colleagues were conducted outdoors, where survival probability was affected not by a single predator, but a community of predators. It is likely that different predators perceive eyespots differently, and thus the parameters that influence the effectiveness of eyespots against predation vary with the predator in question. A fruitful direction for further investigation is to understand the influence of these parameters against different kinds of predators. In addition to quantifying attack frequency and latency, using other communication modalities, such as sound, could furthermore help delineate both hypotheses. Although we did not hear any audible alarm calls eliciting fear, there have been studies with evidence for fear based on alarm calls emitted by predators upon noticing eyespots on prey [18]. Under natural conditions, predation pressure is typically exerted by a predator community, and hence it is possible that different kinds of eyespots and eyespot configurations might be effective in different geographic regions or seasons [28]. This might account for some of the diversity of eyespot patterns found in nature. Similarly, the habitat of the prey may strongly influence the evolution of eyespot-like patterns.

## Conclusion

Our experiments refute specific predictions related to the conspicuousness and eye-mimicry hypotheses, two explanations which have found support to various extents in previous work. In stark contrast to previous reports, our results indicated that neither the size of eyespot-like stimulus nor a close resemblance to the vertebrate eye is critical. Furthermore, moderate asymmetry in shape did not significantly affect the effectiveness of the stimuli. Across the first four experiments, we found that the presence of a pair of patterns on the butterfly model accords the best protection. The importance of pairedness was strongly corroborated in the final experiment, which was designed specifically to test this postulate.

We opine that the observed discord between some of our results and those of previous studies is likely due to different experimental protocols (e.g. indoor *vs* outdoor), extent of resemblance of models to real prey, receiver bias and other factors. Our study underscores the need for experiments based on a variety of predator and experimental systems to understand the functional significance of complex patterns such as eyespots.

## Methods

### Predators and artificial prey

The predators, domestic chickens, comprised ca. 30–45 days old birds hand-reared in the poultry farm of the state-run Kerala State Poultry Development Corporation (KEPCO), Trivandrum, which provided the necessary permits for the experiments. All trials were conducted at KEPCO, during which birds were housed and handled in accordance with national laws, and the Indian National Science Academy guidelines for care and use of animals in scientific research. No bird was injured or killed as part of the study. Experimental protocols were scrutinized and approved by the Department Animal Ethics and Monitoring Committee, School of Biology, IISER Thiruvananthapuram, and the Institutional Animal Ethics Committee. Built adjacent to the farm, the experimental set up comprised a rectangular region of 580 × 480 × 400 mm with caged walls, where the walls and base were covered with cardboard sheets (Additional file 2: Figure S1 i.).

**Figure 4:**
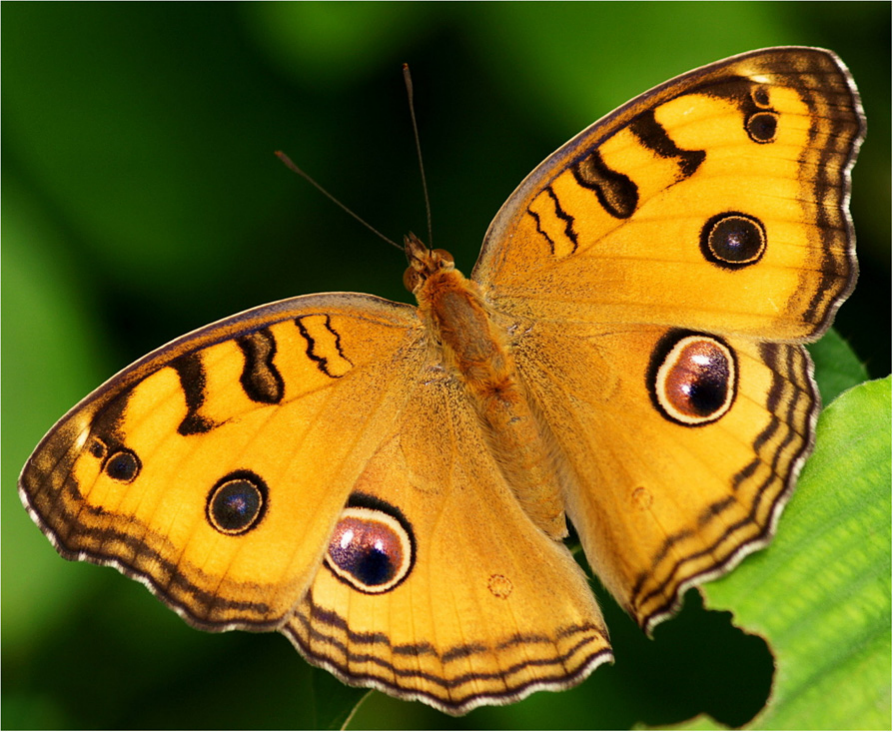
**Photograph of a basking** ***Junonia almana*** **, from which the models were derived.**

Butterfly models were digitally manipulated from a photograph of a real specimen of *J. almana* in its resting position (Figure 4), using GIMP v 2.8 [60] and were printed on matte paper of 60 × 35 mm dimensions, matching the natural wingspan of *J. almana* [61]. In each trial a bird was presented with a choice between two paper models glued onto a beige cardboard of dimensions 250 × 100 mm with a distance of 190 mm between the centres of the butterfly models (Additional file 2: Figure S1 ii.). The patterns along the wing margins were erased, and the wings’ colour was modified to a uniform yellow-ochre, matching the natural dominant wing colour. The shape, size and arrangement of eyespots were manipulated for a series of experiments described in the next section. The birds had been habituated to feed on broken corn, and we therefore decided to use the same in the trials instead of mealworms or other food. Broken corn were placed as a trail to lead the naïve chicken towards the two choices, ending at the cardboard rectangle with the glued paper butterflies. Three broken corn pieces were placed on the mid-body of each model, and a peck on one of the pieces was considered to be an attack.

On the day of the experiment, around 20 chickens were starved for approximately 90 minutes to enhance their motivation to feed. At the onset of each trial, a bird was placed inside the caged setup on the side opposite to the models (Additional file 2: Figure S1 iii.). A trial was terminated either after the bird had attacked both models or after the completion of 5 minutes. The time taken from the bird’s entry into the setup to an attack on each model was recorded using a stopwatch. Observations during pilot trials showed that birds that did not attack within the first 3 minutes were unlikely to do so even after staying in the setup for up to 5 minutes. Hence five minutes was considered an optimal duration for the trials. No bird was used more than once. Within each experiment, the arrangement (i.e. right or left) of the two models with respect to the direction of approach of the bird was randomized.

### Experiment 1

#### i. 0 *vs* 1 eyespot per hindwing ii. 1 *vs* 5 eyespots per hindwing & iii. 0 *vs* 5 eyespots per hindwing

**Figure 5:**
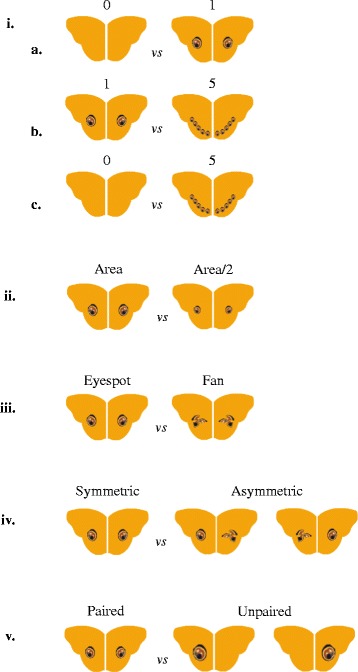
**Models used in the experiment i.** Experiment 1 a. The first test comparing models with no eyespots (0) *vs* one eyespot (1) per hindwing. b. The second test comparing 1 eyespot (1) *vs* 5 eyespots (5) per hindwing. c. The third test comparing no eyespots (0) *vs* 5 eyespots (5) per hindwing. **ii.** Experiment 2. Comparison of models with a pair of natural sized eyespots (Area) *vs* a pair of eyespots of half the area (Area/2) **iii.** Experiment 3. Comparison of models with a pair of natural eyespots (Eyespot) *vs* a pair of fans (Fan) **iv.** Experiment 4. Comparison of models with symmetric eyespots (Symmetric) *vs* a pair of asymmetric patterns comprising an eyespot and a fan (Asymmetric) **v.** Experiment 5. Comparison of models with a pair of natural sized eyespots (Paired) *vs* a single eyespot with twice the area of the natural eyespot (Unpaired).

The conspicuousness hypothesis predicts that the effectiveness of eyespots is determined by their total area. This experiment included three tests. In the first test (0 *vs* 1), 84 birds were given a choice between models with no eyespots and those with 1 eyespot on each hindwing. In the second test (1 *vs* 5), 93 birds were given a choice between models with one eyespot per hindwing and 5 smaller eyespots per hindwing, wherein the total area of the 5 eyespots equalled the area of the single eyespot. In the third test (0 *vs* 5), 89 birds were presented with a choice between models with no eyespots and models with 5 eyespots per hindwing (with the total area being equal to that of the single eyespot). Therefore, the total area of the conspicuous signal is conserved across treatments with eyespots. The eyespots were assumed to be circular; thus the radius of the original eyespot (9 mm diameter) was manipulated to make eyespots of 1/5th the area of the original one. Every day, the birds were divided into three groups for the corresponding tests (Figure 5 i.).

### Prediction

According to the conspicuousness hypothesis, models with eyespots (irrespective of the number) should suffer fewer attacks, and higher attack latency, compared to the model without eyespots. Similarly, there should be no difference in the reaction of the birds upon being presented with equally conspicuous models with 1 or 5 eyespots per hindwing. The eye-mimicry hypothesis predicts that models with a single, large eyespot should suffer least predatory attacks.

### Experiment 2

#### Natural-sized eyespots *vs* eyespots with reduced area

This experiment was designed to test whether a reduction in the area of the conspicuous signal decreases their effectiveness. Birds were presented with a choice between two models, one with two circular spots (diameter = 9 mm) and another with eyespots approximately half the area of the previous (diameter = 6.36 mm) (Figure 5 ii.).

### Prediction

The model with smaller eyespots is predicted to suffer more attacks, and at a lower attack latency according to the conspicuousness hypothesis. There is no strong prediction from the eye-mimicry hypothesis with regard to this experiment, although eye-size might indicate the seriousness of the threat.

### Experiment 3

#### A pair of natural eyespots *vs* a pair of non-eyelike patterns with the same total area

The experiment was designed to investigate the importance of resemblance to eyes. We presented 77 birds with a choice between models with a pair of natural eyespots and ones with a pair of fan-like structures, hereafter referred to as fans, made from dividing the original eyespot into 3 sectors, hence occupying the same total area, yet losing their resemblance to eyes (Figure 5 iii.).

### Prediction

The eye-mimicry hypothesis predicts that the pair of fan-like structures should be attacked more frequently. Whereas, both models, being equally conspicuous, should be attacked similar number of times according to the conspicuousness hypothesis.

### Experiment 4

#### A pair of symmetric eyespots *vs* a pair of asymmetric patterns consisting of an eyespot and a fan

The experiment tested the significance of shape symmetry of eyespots for deterring predators. We presented 109 birds with models containing a shape-symmetric pair of natural eyespots and models with shape-asymmetric, yet equally conspicuous, patterns comprising a natural eyespot and a fan (Figure 5 iv.). The placement of the fan and circular eyespot on the left and right wings was alternated after each trial.

### Prediction

According to the eye-mimicry hypothesis, models bearing shape-symmetric patterns should suffer fewer attacks. Whereas, the conspicuousness hypothesis predicts that both models should suffer similar number of attacks.

### Experiment 5

#### A pair of natural eyespots *vs* a single eyespot with twice the area

This experiment tested whether pairedness, also characteristic of vertebrate eyes, is important for intimidation. The reaction of 75 naïve birds when offered a choice between models with two natural eyespots (diameter = 9 mm) and models with an equally conspicuous singular eyespot with twice the area of one natural eyespot (diameter = 12.728 mm) on either one of the hindwings, was tested. The placement of the single eyespot on the right or left wing was alternated (Figure 5 v.).

### Prediction

The eye-mimicry hypothesis predicts that a pair of eyespots should induce more avoidance. On the other hand, the conspicuousness hypothesis predicts that both treatments should be equally preferred by birds.

### Statistical analyses

All statistical analyses were done in R v 2.9.2 [62]. An exact binomial test with equal *a priori* probability for each choice was performed to test whether birds preferred to first attack one butterfly model to the other. The paired Wilcoxon signed-rank test was conducted to compare the time taken by the bird to attack one model versus the time taken to attack the second one. To compare the latency to attack the second model after the first, a Mann–Whitney *U* Test was performed.

## Availability of supporting data

The data set supporting the results of this article is available in the Dryad repository, doi:10.5061/dryad.390t3 [63].

## Additional files

Additional file 1: Figure S2. Boxplots describing the latencies between the first and second attack (in seconds) of the birds. The dark horizontal line within the box represents the median with the box denoting the first and third quartiles and the whiskers being the minimum and maximum values observed. When presented with **i.** No *vs* 1 eyespot/hindwing (0: m = 20 s, IQR = 51 s; 1: m = 5.5, IQR = 11.25 s); **ii.** 1 *vs* 5 eyespots/hindwing (1: m = 12 s; IQR = 55 s; 5: m = 20 s, IQR = 57 s); **iii.** 0 *vs* 5 eyespots/hindwing (0: m = 10 s, IQR = 35.75 s; 5: m = 6 s, IQR =17 s); **iv.** Area *vs* Area/2 eyespots/hindwing (Area: m = 11 s, IQR = 62.5 s; Area/2: m = 5 s, IQR = 33.25 s); **v.** Eyespot *vs* Fan-like eyespots/hindwing (Eyespot: m = 10 s, IQR = 39.25 s; Fan: m = 5 s, IQR = 50 s); **vi.** Symmetric *vs* Asymmetric eyespots/hindwing (Symmetric: m = 9.5 s, IQR = 25 s; Asymmetric: m = 9.5 s, IQR = 43.75 s); **vii.** Paired *vs* Unpaired eyespots/hindwing (Paired: m = 15 s, IQR = 44 s; Unpaired: m = 10.5 s, IQR = 44.75 s). The latency was significantly higher only when the birds attacked the model with 1 eyespot/hindwing after attacking model with none (Mann–Whitney *U* Test: W = 942, P = 0.0276). Additional file 2: Figure S1. The experimental setup **i.** Photograph of the caged setup with the presented models. **ii.** The butterfly models glued 190 mm apart on a beige cardboard in one of the experiments. **iii.** Photograph of a chicken approaching models in the setup.
