# Supplementary figures and images for: What makes eyespots intimidating–the importance of pairedness
Source: BMC Evol Biol. 2015 Mar 9;15:34. doi: 10.1186/s12862-015-0307-3 (PMC4374370; doi:10.1186/s12862-015-0307-3)

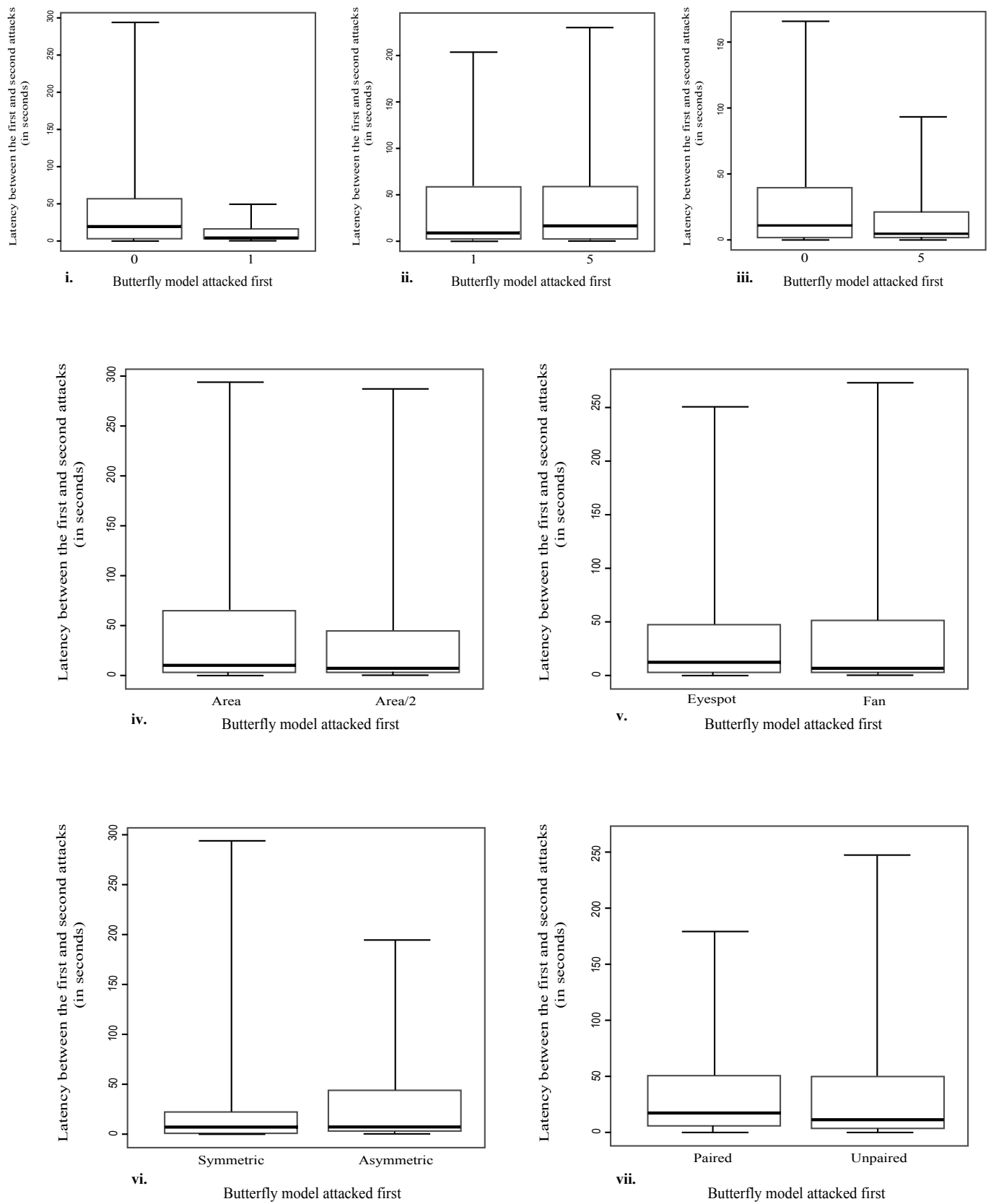

**Figure S2**

Supplement: Additional file 1: Figure S2. — Boxplots describing the latencies between the first and second attack (in seconds) of the birds. The dark horizontal line within the box represents the median with the box denoting the first and third quartiles and the whiskers being the minimum and maximum values observed. When presented with i. No vs 1 eyespot/hindwing (0: m = 20 s, IQR = 51 s; 1: m = 5.5, IQR = 11.25 s); ii. 1 vs 5 eyespots/hindwing (1: m = 12 s; IQR = 55 s; 5: m = 20 s, IQR = 57 s); iii. 0 vs 5 eyespots/hindwing (0: m = 10 s, IQR = 35.75 s; 5: m = 6 s, IQR =17 s); iv. Area vs Area/2 eyespots/hindwing (Area: m = 11 s, IQR = 62.5 s; Area/2: m = 5 s, IQR = 33.25 s); v. Eyespot vs Fan-like eyespots/hindwing (Eyespot: m = 10 s, IQR = 39.25 s; Fan: m = 5 s, IQR = 50 s); vi. Symmetric vs Asymmetric eyespots/hindwing (Symmetric: m = 9.5 s, IQR = 25 s; Asymmetric: m = 9.5 s, IQR = 43.75 s); vii. Paired vs Unpaired eyespots/hindwing (Paired: m = 15 s, IQR = 44 s; Unpaired: m = 10.5 s, IQR = 44.75 s). The latency was significantly higher only when the birds attacked the model with 1 eyespot/hindwing after attacking model with none (Mann–Whitney U Test: W = 942, P = 0.0276). [file 12862_2015_307_MOESM1_ESM.pdf]

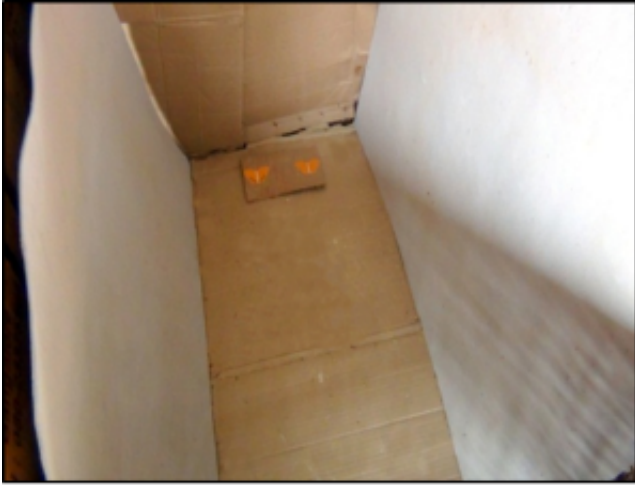

i.

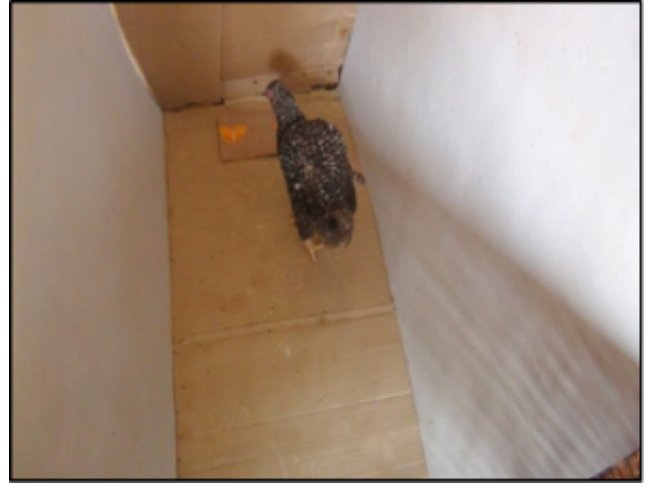

iii.

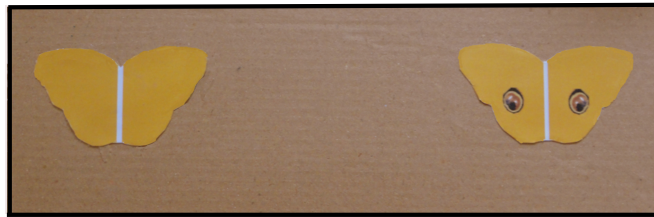

ii.

Figure S1

Supplement: Additional file 2: Figure S1. — The experimental setup i. Photograph of the caged setup with the presented models. ii. The butterfly models glued 190 mm apart on a beige cardboard in one of the experiments. iii. Photograph of a chicken approaching models in the setup. [file 12862_2015_307_MOESM2_ESM.pdf]
